# Targeted Therapeutic Strategies in the Battle Against Pathogenic Bacteria

**DOI:** 10.3389/fphar.2021.673239

**Published:** 2021-05-12

**Authors:** Bingqing Yang, Dan Fang, Qingyan Lv, Zhiqiang Wang, Yuan Liu

**Affiliations:** ^1^ College of Veterinary Medicine, Yangzhou University, Yangzhou, China; ^2^ Joint International Research Laboratory of Agriculture and Agri-Product Safety, The Ministry of Education of China, Yangzhou University, Yangzhou, China; ^3^ Jiangsu Co-Innovation Center for Prevention and Control of Important Animal Infectious Diseases and Zoonoses, Yangzhou University, Yangzhou, China; ^4^ Institute of Comparative Medicine, Yangzhou University, Yangzhou, China

**Keywords:** targeted therapeutic strategies, pathogenic bacteria, probiotics, phage, CRISPR-Cas9

## Abstract

The emergence and rapid spread of antibiotic resistance in pathogenic bacteria constitute a global threat for public health. Despite ongoing efforts to confront this crisis, the pace of finding new potent antimicrobials is far slower than the evolution of drug resistance. The abuse of broad-spectrum antibiotics not only accelerates the formation of resistance but also imposes a burden on the intestinal microbiota, which acts a critical role in human homeostasis. As such, innovative therapeutic strategies with precision are pressingly warranted and highly anticipated. Recently, target therapies have achieved some breakthroughs by the aid of modern technology. In this review, we provide an insightful illustration of current and future medical targeted strategies, including narrow-spectrum agents, engineered probiotics, nanotechnology, phage therapy, and CRISPR-Cas9 technology. We discuss the recent advances and potential hurdles of these strategies. Meanwhile, the possibilities to mitigate the spread of resistance in these approaches are also mentioned. Altogether, a better understanding of the advantages, disadvantages, and mechanisms of action of these targeted therapies will be conducive to broadening our horizons and optimizing the existing antibacterial approaches.

## Introduction

The past few centuries have witnessed the ceaseless battles between pathogenic bacteria and antibiotics. Although the proceeding of antibiotic discovery is never at a standstill, bacteria have numerous strategies to escape from antibiotic killing and even upgrade to the so-called superbug. Typical examples lie in the appearance of multidrug-resistant (MDR) bacteria, such as the notorious vancomycin-resistant enterococci (VRE) (Gonzales et al., 2001) and extended spectrum beta-lactamase (ESBL)-producing Enterobacteriaceae (Paterson et al., 2003). Besides, the globalization of resistance is without doubt making things worse, as is reflected in the dissemination and prevalence of New Delhi metallo-beta-lactamase-1 (NDM-1) from India to Pakistan, the United States, Canada, Japan, and the United Kingdom (Walsh et al., 2011). As the European Centre for Disease Prevention and Control (ECDC) revealed, there were approximately 25,000 people dying from infections caused by MDR bacteria every year just in Europe (Carlet and Mainardi, 2012). A recent report published by the United Kingdom government also predicted that the global death toll would increase to 10 million by 2050 if new antimicrobial strategies were not discovered (O'Neill, 2016). Evidently, now is the time people are stepping into the “post-antibiotic” era, in which resistance crisis appears, and many antimicrobials are no longer effective.

The rapid emergence and evolution of antibiotic resistance can be attributed to many reasons, such as inappropriate treatment regimen and supplement in feed as animal growth promoter, among which the abuse of broad-spectrum antimicrobials plays a dominant role. In particular, owing to the lack of rapid diagnosis of pathogens, broad-spectrum antibiotics are empirically employed in nosocomial infections as countermeasures. However, their indiscriminate killing modes and the accompanying selective pressure really promote the emergence of antibiotic resistance. To be specific, antibiotic treatment kills all sensitive bacteria, while a part of them gradually acquire resistant mutations and survive. Additionally, broad-spectrum antimicrobials impose detrimental effects on the structure and diversity of intestinal microbiota. However, it is widely acknowledged that a balanced intestinal microbiota plays an important role in human health. For one thing, it helps to absorb nutrition and maintain the regular intestinal motility. For another thing, gut microbiota can inhibit the invasion of pathogenic bacteria or opportunistic bacteria through space-occupying protection and the production of substances such as bacteriocin, organic acid, and hydrogen peroxide. Without the restriction of normal microbiota, the overgrowth of drug-resistant bacteria will inevitably lead to serious dysbacteriosis like pseudomembranous colitis (PMC), which is often known as the so-called off-target effects (Blaser, 2016). Sometimes, the side effects of longtime antibiotic intake like hyperspasmia, coma, tinnitus, nausea, and renal insufficiency also bring about much trouble to the patients.

Given all of the above, the exploitation and application of antimicrobials are always necessary to combat against the increasingly serious resistance crisis. Under this premise, hunting for more precise and targeted antimicrobial strategies capable of protecting our normal microbiota seems more significant. At present, with the swift development and innovation of modern science and technology, a great number of inspirations and assumptions have been put forward and realized in precise sterilization. Herein, we discuss recent advances and the prospects of targeted therapeutic strategies in the fight against pathogenic bacteria, including narrow-spectrum agents, engineered probiotics, nanotechnology, phage therapy, and CRISPR-Cas9 technology.

## Narrow-Spectrum Antimicrobial Agents

The broad-spectrum antimicrobial agents are empirically employed in clinical settings because the long time to perform bacteria isolation and identification may lead to treatment delay and condition deterioration. Also, broad-spectrum agents possess the ability to kill an extensive scope of bacteria, which is suitable for clinically common mixed infections. However, the indiscriminate killing mode of broad-spectrum agents results in the death of all bacteria, thus disturbing the microbiota balance and even inducing the superinfection. For instance, 2- to 7-year-old Finnish children treated with macrolide showed a long-lasting shift in microbiota composition and metabolism, including increase abundance of Bacteroidetes and Proteobacteria, decrease in bile-salt hydrolase, and increase in macrolide resistance (Korpela et al., 2016). On the contrary, more pointed narrow-spectrum antimicrobial agents, including narrow-spectrum antibiotics, antibacterial peptides (AMPs), and lysins, show the possibility to cope with this dilemma and achieve targeted therapy.

### Narrow-Spectrum Antibiotics

Since the discovery of penicillin, a series of antibiotics have been exploited from soil, marine, insect, human, and plant. They perform killing or inhibiting functions based on different targets and different bacterial phases. As the most dominant traditional antimicrobial agent, antibiotics never let us down until nowadays although there is a slowdown recently in finding new sources. Among these rich resources, narrow-spectrum antibiotics outstand due to their precise modes of action without additional effects (Table 1).

**TABLE 1:** Sources, spectrums, and modes of action of representative narrow-spectrum antibiotics.

Antibiotics	Sources	Antibacterial spectrums	Modes of action	References
Fidaxomicin	*Dactylosporangium aurantiacum*	*C. difficile*	Inhibits RNA polymerase	Sears et al. (2013)
Ridinilazole	Unknown	*C. difficile*	Enhances the preservation of microbiota-dependent bile acids	Qian et al. (2020)
Teixobactin	Uncultured organisms	Gram-positive bacteria	Inhibit the synthesis of cell wall	Ling et al. (2015)
A thiochromenone antibiotic	*Pseudomonas* quinolone signal	*Moraxella catarrhalis*	Inhibits a target in the primary energy metabolism	Szamosvári et al. (2019)
Thuricin Z	*Bacillus thuringiensis*	*B. cereus*	Causes membrane permeabilization	Mo et al. (2019)
Lysocin E	Soil bacteria	Bacteria with menaquinone	Loss of membrane potential	Hamamoto et al. (2015)
Lugdunin	Human-associated bacteria	Gram-positive bacteria	Breakdown of bacterial energy resources	Zipperer et al. (2016)
Corbomycin and complestatin	*Actinomycetes*	Gram-positive bacteria	Inhibits peptidoglycan remodeling	Culp et al. (2020)
Darobactin	Photorhabdus symbionts of entomopathogenic nematode microbiomes	Gram-negative bacteria	Disrupts the formation of a functional outer membrane	Imai et al. (2019)
G0775	Arylomycins	Gram-negative bacteria	Inhibits bacterial type I signal peptidase	Smith et al. (2018)
Ilamycins E1/E2	*Streptomyces atratus* SCSIO ZH16	*M. tuberculosis*	Unknown	Ma et al. (2017)
Novel griselimycins	*Streptomyces*	*M. tuberculosis*	Inhibits the DNA polymerase sliding clamp dnan	Kling et al. (2015)
Linezolid	Synthetic antibiotic	Gram-positive bacteria	Inhibits bacterial protein synthesis	Hashemian et al. (2018)
Daptomycin	*S. reseosporus*	Gram-positive bacteria	Disrupts cell membrane	Heidary et al. (2018)
Microcin B17	Bacteria with pMccB17	Gammaproteobacteria	Inhibits DNA replication	Collin and Maxwell (2019)
Plantazolicin	*Bacillus methylotrophicus* FZB42 and *Bacillus pumilus*	*B. anthracis*	Disrupts cell membrane	Molohon et al. (2016)
NVB302	Deoxyactagardine B	*C. difficile*	Inhibits cell wall biosynthesis	Petrosillo et al. (2018)

In fact, some of them are still based on previously reported targets such as cell wall synthesis, membrane permeability, protein synthesis, and DNA transcription. For example, fidaxomicin, derived from *Dactylosporangium aurantiacum*, possessed distinctive bactericidal activity against *Clostridioides difficile* but minimal activity against the original gut microbiota (Sears et al., 2013). As an RNA polymerase inhibitor, it was different from rifampicin as it inhibited RNA polymerase in the early stage of transcription, even prior to initiation of mRNA synthesis. Thuricin Z, a novel sactibiotic from *Bacillus thuringiensis*, was also reported to be capable of causing membrane permeabilization of *Bacillus cereus* with specificity (Mo et al., 2019). Another exciting incident was the discovery of soil-derived teixobactin, which was only active against Gram-positive bacteria (Ling et al., 2015). It could inhibit the synthesis of cell wall by binding to a highly conserved motif of lipid II (precursor of peptidoglycan) and lipid III (precursor of teichoic acid) without detectable resistance.

Additionally, the appearance of new targets or new mechanisms seems more attractive. For example, an antibiotic termed ridinilazole was found efficient in the treatment of *C. difficile* infections through enhancing the preservation of microbiota-dependent bile acid metabolome without interfering with the commensal microbiota (Qian et al., 2020). Moreover, antibiotics with killing modes based on energy metabolism inhibition show a novel pipeline for drug development. A thiochromenone antibiotic derived from the *Pseudomonas* quinolone signal (PQS) exhibited highly potent antibiotic activity against *Moraxella catarrhalis* likely by inhibiting a target in the primary energy metabolism (Szamosvári et al., 2019). Consistently, cellular ATP concentrations in *M. catarrhalis* dropped significantly after exposure to this compound. However, the specific targets of this thiochromenone antibiotic are still unclear. Similarly, lugdunin was reported to be related to the swift breakdown of bacterial energy resources as it ceased the incorporation of radioactive precursors of DNA, RNA, protein, and cell wall under low concentrations nearly at the meantime (Zipperer et al., 2016). It is noteworthy that lugdunin was capable of coping with a series of Gram-positive bacteria, especially the notorious methicillin-resistant *S. aureus* (MRSA) and the VRE isolates.

Together, these precise antibiotics harbor few and critical sites of action that closely take part in bacterial survival and reproduction. They can be more suitable, more efficient, and would not induce the subsequent severe infections. One of the principles of antibiotic use is the prioritization of narrow-spectrum antibiotic, especially when we have figured out the specific pathogenic bacteria. It is good news for clinical treatment but indeed places a higher expectation on rapid diagnosis.

### Narrow-Spectrum AMPs

Antimicrobial peptides (AMPs), also called host-defense peptides (HDPs), are important components of the innate immune system (Lazzaro et al., 2020). As their modes of action primarily depend on the mechanism involving electrostatic interactions between their cationic domains and negatively charged bacterial cell surface, AMPs have a lower likelihood to induce host toxicity because eukaryotic cell membrane is electrically neutral (Jenssen et al., 2006). In this regard, AMPs are not prone to induce drug resistance, thus showing broad prospects to perform as ideal antibiotic alternatives in this resistance era (Liu et al., 2021). More importantly, AMPs with targeted activity turn out to be good choices for precise killing. For instance, the AMP thanatin from *Podisus maculiventris* was found to reverse carbapenem resistance in NDM-1–producing bacteria by dual mechanisms (Ma et al., 2019), that is, disrupting the outer membrane by competitively displacing divalent cations and inhibiting the enzymatic activity of NDM-1 by displacing zinc ions from the active site. ZY4, a cyclic peptide, not only induced membrane permeabilization in bacteria with low frequency of resistance but also showed supreme potency in coping with persister cells (Mwangi et al., 2019). It had been confirmed to be able to combat with multidrug-resistant *P. aeruginosa* and *A. baumannii* infections potently than *Bacillus subtilis* and *S. aureus*.

Except membrane disruption, AMPs also play a part in several intracellular processes. In a previous work, it was reported that sublethal concentrations of P-Der from pleurocidin or frog dermaseptin could inhibit macromolecular synthesis in *Escherichia coli* (Patrzykat et al., 2002)*.* Additionally, targets relevant to the formation of structural components are also worthy to be noted. For instance, a non-ribosomal lipopeptide tridecaptin A1 (TriA1) produced by *Bacillus* and *Paenibacillus* species exerted antibacterial activity against Gram-negative bacteria by binding to lipid II on the inner membrane and disrupting the proton motive force (Cochrane et al., 2016). Besides, strategies based on database-filtering technology provided us inspiration to generate ideal, short, specific, and effective AMPs. For instance, the potential peptides F1 and F4 from the antimicrobial peptide database (APD) with an α-helical symmetrical structure displayed short, safe, and stable activity against Gram-negative pathogens such as *E. coli*, *S. pullorum*, and *P. aeruginosa* (Chou et al., 2019)*.* However, their activities against Gram-positive bacteria were very poor as reflected in the high MIC values in *S. epidermidis* and *P. aeruginosa*.

Until recently, although peptide antibiotics such as polymyxin B, colistin, and daptomycin have been put into clinical use for decades, doubts and criticisms constantly exist due to their incomplete success with dose-dependent toxicity and short half-life *in vivo*. So, after sophisticated designing and screening, AMPs with activities still need to undergo tests in terms of protease stability and cytoxicity. In this case, structural modification such as lipophilic fatty acid chain removal (Liu et al., 2017) and strategies providing protease resistance such as amino acid type replacement (Braunstein et al., 2004) deserve serious consideration.

### Narrow-Spectrum Lysins

At the end of the lytic cycle of bacteriophages, lysins will be released and accumulated in the cytoplasm (Fischetti, 2008). The nature of lysin is enzyme, which is composed of two parts: the catalytic domain in the *N*-terminal region and the binding domain in the *C*-terminal region. The former exhibits catalytic activity, and the latter has specificity for molecules existing in the host cell wall. Considering this mode of action, we may conclude their excellent activities against Gram-positive bacteria with cell wall and the inefficiency toward Gram-negative bacteria possessing an impermeable outer membrane. However, based on comprehensive understanding, lysins are indeed potent targeted antimicrobial agents owing to their high specificity, low frequency of resistance, and rich sources (Sharma U. et al., 2018). First and foremost, the values of lysins in infection control, especially in aquaculture and stock farming, have been partly acknowledged. One typical example is the lysin PlyC produced by phage C1, which had distinctive and exclusive therapeutic activity against *Streptococcus* sp., whose swift propagation has confused many horse owners (McGowan et al., 2012). Likewise, Cpl-1 and Pal, lysins isolated from active bacteriophages against *Streptococcus pneumoniae*, targeted the same host but exhibited different catalytic activities (Jado et al., 2003). It is noteworthy that this synergy mechanism indeed reduced the emergence of resistance to lysins. Additionally, working in coordination with oxacillina, the chimeric lysin Clys, synthesized by the fusion of the *N*-terminal domain of phage Twort lysin, and the *C*-terminal domain of the phage phiNM3 lysin, exhibited good protection against MRSA in *in vivo* models (Daniel et al., 2010). It is without doubt an imperative work as MRSA is responsible for various skin and soft-tissue infections and symptoms associated with dairy cow mastitis. Another lysin with the same target is CF-301, which was specialized in disrupting *S. aureus* biofilms (Schuch et al., 2017).

The flooding of lysins is also good news for all kinds of nosocomial infections. PlyE146, a novel lysin derived from phage genome–based screening, exhibited supreme bactericidal activity and promising therapeutic efficacy against *E. coli*, *P. aeruginosa*, and *A. baumannii* (Larpin et al., 2018). It is no exaggeration to say that these pathogens really suffer numerous patients racked with septicemia, peritonitis, and urinary-tract infections. However, in other tested strains such as *K. pneumoniae*, *S. enterica*, *S. aureus*, and *S. mitis*, no antibacterial activity was observed.

Food industry also benefits a lot during these endeavors. Ply3626 was reported to display potent activity against *Clostridium perfrigens*, a common pathogen closely related to food poisoning (Zimmer et al., 2002). And as for Ply511, when it was cloned in and secreted by *L. lactis*, it could inhibit the pathogenic *Listeria monocytogenes* during the production of dairy starter cultures, thus showing bio-preservation properties (Gaeng et al., 2000).

Although lysins released by phages are somewhat ignored or underestimated, they still have a place in antibacterial treatment. As enzyme preparations, they are relatively safe to the host and easy to control. Also, through genetic engineering technology, catalytic domains and binding domains of different lysins can be connected to form chimeric lysins with high bactericidal activities (Kim et al., 2019). Considering its rich resources, phage lysin is regarded as an ideal agent with potential and distinction in preventing and controlling bacterial infections.

## Engineered Probiotics

Due to extensive use of antibiotics, many infectious diseases have been cured, but a series of side effects spring up, such as the increasing sensitivity. In this case, drugs with auxiliary and complementary effects for antibiotics are urgently warranted. Probiotic was first defined by Lilly and Stillwell to describe substances produced by protozoa to stimulate the growth of another organism (Lilly and Stillwell, 1965). Long before, their existences in fermented milk were regarded as the secret of good health and longevity in Bulgaria. Thereafter, their efficacies in inhibiting the growth of several pathogenic bacteria and preventing antibiotic-associated diarrhea were gradually found. Hence, they were described as live nonpathogenic bacterial species administered to provide beneficial effects to host through regulating microbial balance, particularly in the gastrointestinal tract (Williams, 2010). Within these decades, advancements in synthetic biology also render it possible to achieve targeted antimicrobial therapy through designing sophisticated engineered probiotics with desired characteristics and functions. Herein, we will discuss the reconstruction and utilization of probiotics in targeted therapy from two main angles.

### Producing or Transporting Specific Antimicrobial Molecules

On the one hand, engineered probiotics can serve as optimal vectors to produce or transport antibacterial molecules targeting specific pathogens. The combination of probiotics and targeted peptides is one of good choices. One example is *L. lactis* IL1403, a reconstructed *L. lactis* that expressed antimicrobial peptides aiming at Gram-negative pathogenic bacteria (Volzing et al., 2013)*.* Herein, synthetic peptides A3APO and alyteserin were sorted out due to their supreme performances among numerous candidates in strong activity against *E. coli* and *Salmonella* while lower activity against *L. lactis.* Another example lies in *E. coli* Nissle 1917, which was designed to express and secrete three antimicrobial peptides: Enterocin A, Enterocin B from *E. faecalis*, and Hiracin JM79 from *E. hirae*. They all processed distinctive activity against both *E. faecium* and *E. faecalis*, the chief culprits to blame for VRE infections (Geldart et al., 2018). In addition, probiotics producing functional fatty acids are also promising. For instance, Peng et al. (2018) increased the production of conjugated linoleic acids (CLAs) by overexpressing the myosin cross-reactive antigen gene (*mcra*) in *Lactobacillus casei* (LC) to specifically inhibit *Salmonella enterica* serovar Typhimurium (ST) and enterohaemorrhagic *Escherichia coli* (EHEC) in gut intestinal infections. Moreover, enzymes with degradation activity can also be employed. In a recent research, Chappell and Nair (2020) rendered two lactobacilli the ability to secrete enzymes degrading *P. aeruginosa* biofilms, and the best-designed engineered lactic acid bacteria producing PA14-derived enzyme (PelA_h_) exhibited a high degrading rate up to 85%.

### Bacteria-Specific Sense-and-Kill System

On the other hand, engineered probiotics are designed to sense the specific metabolites produced by pathogens and make responses. The involvement of probiotics indeed makes sense as it is different from exogenously inserted plasmids which inevitably bring about additional burden to the host. The existence of probiotics makes up for the defects of their commensal bacteria or provides some advantages, which explains hosts’ allowance of their consistent existences. However, this mechanism somewhat has specificity as it relies on the quorum-sensing phenomenon or exclusive properties of several pathogens to utilize or secrete some compounds in competitive environment. In this section, we select some typical pathogens to describe at length, owing to their non-negligible perniciousness, that is, *P. aeruginosa*, *Salmonella*, and *V. cholerae.*

#### *P. aeruginosa* Infections

**FIGURE 1:**
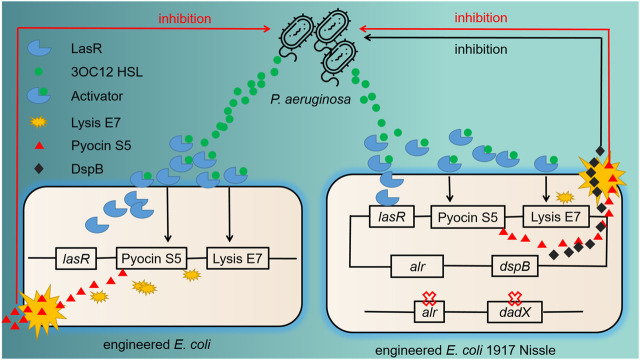
Previous and further improved antimicrobial engineered probiotics through quorum sensing. The left part shows the schematic of the “sense-and-kill” system. 3OC12HSL, the quorum-sensing molecule released by *P. aeruginosa*, binds with LasR to form LasR–3OC12HSL complex. This complex thereafter activates the production of E7 lysis protein and S5 pyocin. When the accumulation of E7 protein is enough to lead to the cell lysis, Pyocin S5 is released into the exogenous environment and kills *P. aeruginosa*. The right part shows the improvement of this system. The chromosomal deletion of *alr* and *dadX* genes in *E. coli* Nissle promotes the retention of plasmid carrying *alr* gene. An evident difference of the killing part is the addition of *dspB*, which encodes an anti-biofilm enzyme with enhanced inhibition efficacy.

*P. aeruginosa*, a Gram-negative pathogen, can cause severe infections as it harbors natural resistance feature, biofilm-forming abilities, and effective efflux systems (Chang et al., 2005). For combating *P. aeruginosa* infections, Saeidi et al. designed engineered *E. coli* with “sense-and-kill” system dependent on quorum sensing (Figure 1). In this system, the production of LasR was activated by *P. aeruginosa* quorum-sensing molecule *N*-acyl-homoserine lactone (AHL), which therefore opened up the expression of E7 lysis protein and pyocin S5. Once the E7 protein reached the threshold concentration of chassis lysis, the accumulated S5 was then released into the exogenous environment and killed *P. aeruginos*a efficiently (Saeidi et al., 2011). Thereafter, this system was optimized in *E. coli* Nissle 1917 with the addition of dispersin B (DspB), a new anti-biofilm enzyme produced by *Actinobacillus actinomycetemcomitans* with glycosyl hydrolase activity. Evaluation of the engineered probiotic strain in a *C. elegans* infection model and a mouse infection model showed good treatment results (Hwang et al., 2017).

#### *Salmonella* Infections

As a common bacterium of intestinal infection, *Salmonella* poses a threat to the health of human and animals such as livestock, rodents, and poultry (Mastroeni and Sheppard, 2004). Previous research showed that microcin H47 (MccH47), a chromosome-encoded peptide with high molecular mass from *E. coli* strain H47, inhibited the growth of *Salmonella in vitro* potently (Laviña et al., 1990). Meanwhile, the gene products of *ttr* operon (ttrRSBCA) enabled *Salmonella* species to utilize tetrathionate as an electron acceptor for respiration and outcompete other bacteria with a growth advantage. Tetrathionate is usually generated as the reaction of luminal thiosulfate and reactive oxygen species produced during gut inflammation (Winter et al., 2010). Therefore, Palmer et al. (2018) successfully reconstructed *E. coli* strain Nissle 1917 with a plasmid-based MccH47 induction system and tetrathionate sensing system to cope with *Salmonella* infections.

#### *V. cholerae* Infections

**FIGURE 2:**
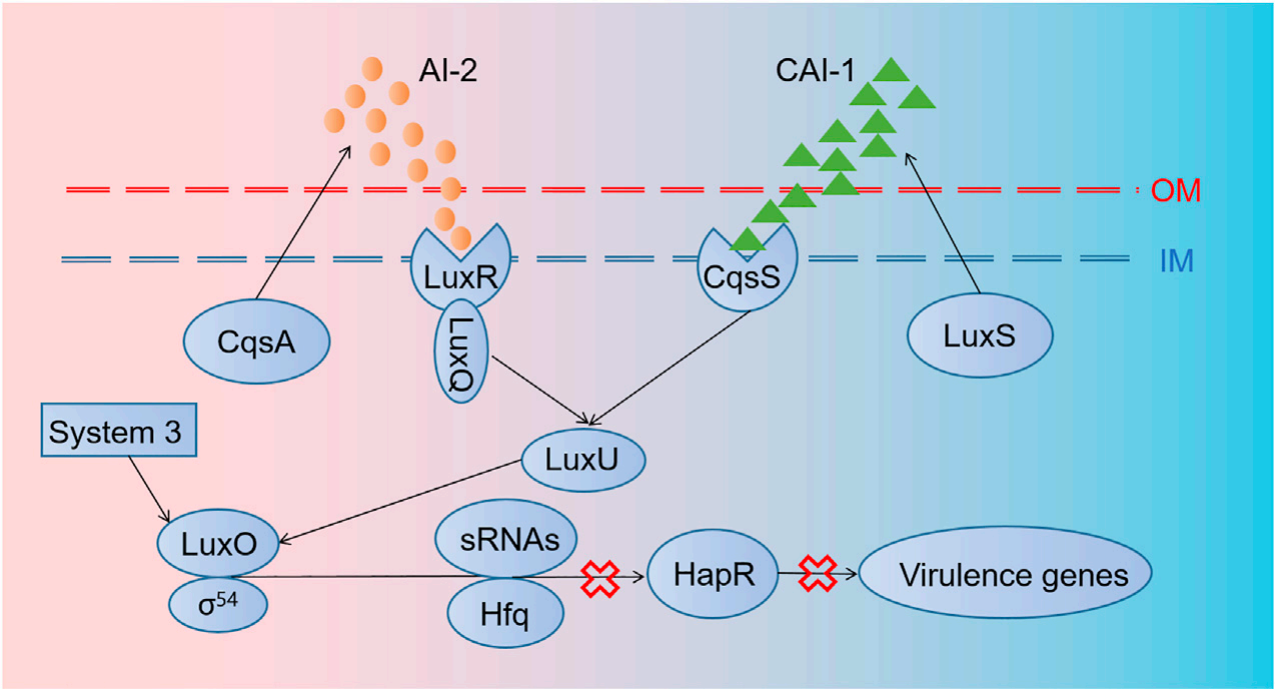
Regulation mechanisms of quorum sensing in *V. cholerae.* The parallel QS system of *V. cholerae* possesses AI-2/LuxPQ and CAI-1/CqsS systems, as well as an unidentified third circuit that acts through LuxO. *cqsA* and *luxS* genes encode proteins producing AI-2 and CAI-1. Thereafter, AI-2 and CAI-1 are detected and captured by LuxRQ complex and CqsS, respectively, leading to the activation of LuxU and LuxO later. Then, with the aid of σ^54^, sRNAs and their chaperones Hfq are transformed into sRNAs–Hfq complex, which inhibits the transcription of HapR mRNAs, thus blocking the forming of HapR, a transcription factor relevant to the expression of some virulence genes.

*V. cholerae* causes millions of deaths from cholera per year as it induces severe watery diarrhea by colonizing in the small intestine and producing toxins (Ali et al., 2015). The parallel QS system of *V. cholerae* possesses AI-2/LuxPQ and CAI-1/CqsS systems as well as an unidentified third circuit that acts through LuxO [Figure 2; Miller et al. (2002)]. They work together to coordinate gene expression when *V. cholerae* populations reach higher concentrations. In order to fight against *V. cholerae*, Duan and March (2010) employed an engineered commensal bacteria *E. coli* Nissle 1917 expressing AI-2 and CAI-1 simultaneously, which could disturb bacterial communication to inhibit *V. cholerae* virulence. Based on the same mechanism, Mao et al. (2018) designed a cholera-sensing and reporting system by engineered *L. lactis* through developing a receptor in *L. lactis* to detect CAI-1 from *V. cholerae* (Mao et al., 2018). Then, *L. lactis* could sharply reduce intestinal *V. cholerae* load and get an improved survival rate in the infected infant mice model by producing lactic acid. Another strategy was to compete with intestinal receptor binding with cholera toxins through designing a recombinant bacterium expressing glycosyltransferase genes, which terminated the production of a chimeric lipopolysaccharide (Focareta et al., 2006).

Hopefully, except bacteria from the genera *Lactobacillus* and nonpathogenic strains of *E. coli* as we discussed above*,* the yeast *Saccharomyces boulardii*, *Bacillus* spp, and bacteria of *Bifidobacterium* can also be exploited, serving as excellent candidates (Kotzampassi and Giamarellos-Bourboulis, 2012). The mechanism of probiotic can be concluded as a first recovery of microbiota balance and the following outgrowth of probiotics to cope with pathogenic bacteria. Usually, their modes of action are direct, while there are also some interactions between bacteria and host immune defenses (Isolauri et al., 2002).

## Nanomaterial-Based Targeted Therapy

**FIGURE 3:**
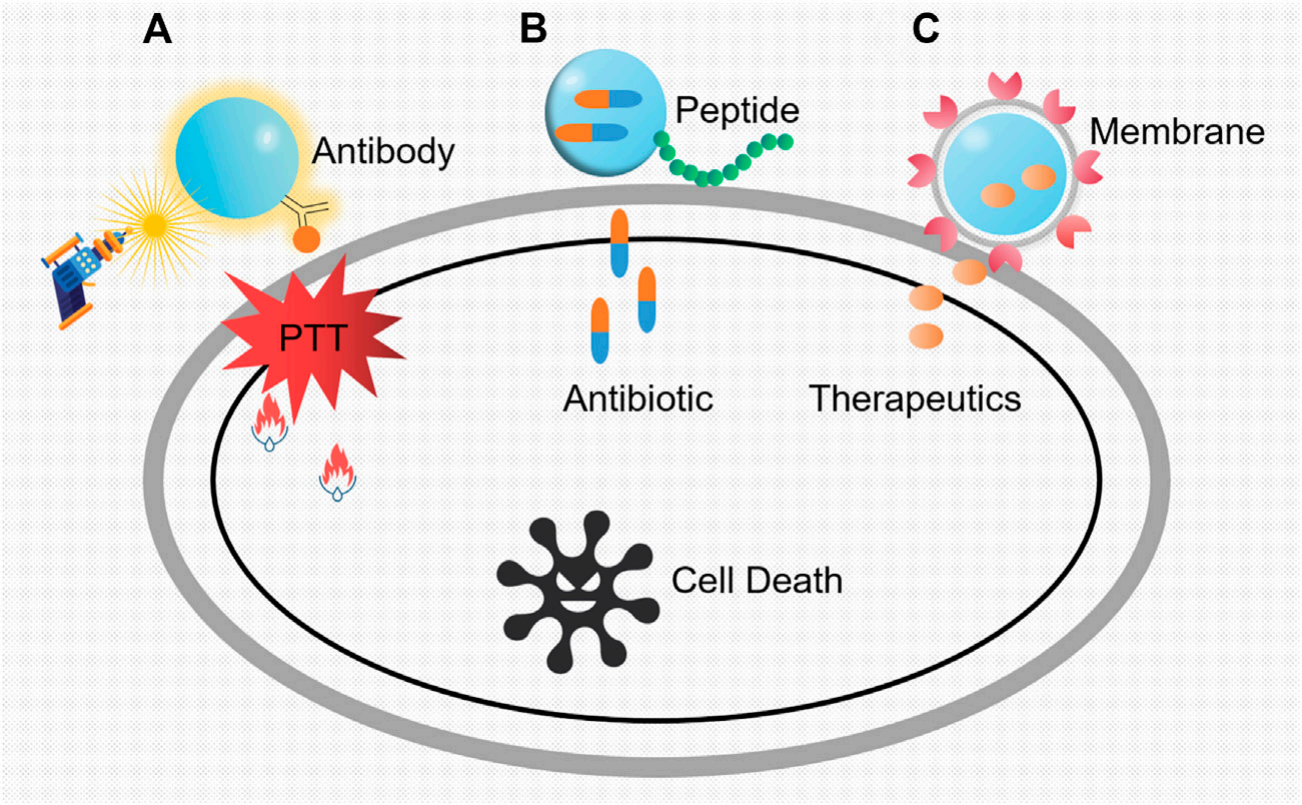
Three main modes of nanoparticles as potent vehicles to target pathogens. **(A)** Killing mechanisms of nanoparticles loaded with antibody specific to the pathogenic bacteria. The constant exposure of laser irradiation was transformed into heat, which leads to the regional cell damage and the inevitable cell death. PTT refers to photothermal therapy. **(B)** Antibiotic-loaded nanoparticles with targeted peptides. After the identification of the specific bacterial region, the shell breaks and the inside antibiotic releases. **(C)** The action modes of nanoparticles coated with vesicles from platelet membrane. Owing to the pretreatment with specific bacteria, markers on vesicle membranes will conduct recognition, and the target bacteria will be killed by the subsequent release of the therapeutics.

Despite the emergence of a good many antimicrobial agents, the mortality of bacterial infections, especially in nosocomial settings, keeps high constantly. As we know, traditional “naked drug” delivery is the process of delivering therapeutic molecules to the targeted cells or tissues. It has relatively low efficacy as it goes through the experience of administration, distribution, absorption, and elimination. During these processes, effective drug only accounts for a little part of the total as restrictions *in vivo* and renal clearance. Hence, nanoparticle-aided systems (nanotechnology) outstand with high transport efficacy, realizing prolonged and sustained release of drugs and their maximized plasma half-life. In this section, concluding from various specialized designs, we briefly illustrate the interactive modes of nanoparticles with antibody, peptide, membrane, and other materials in targeted therapy against pathogens **(** Figure 3).

### Antibody-Based Targeted Nanoparticles

Antibody is one kind of immunoglobulin that can specifically bind with antigen, harboring abilities such as neutralizing toxins, producing membrane attack complex, and mediating type I hypersensitivity. Besides these, antibodies can be ideal antimicrobial agents by means of virulence factor neutralization, enhancement of opsonophagocytic uptake and killing (OPK), and complement-mediated bacterial lysis (Nagy et al., 2017). Through the specific combination of antigens and antibodies, metal nanoparticles with antibodies can target pathogens and perform bactericidal activities with intrinsic characteristic such as photothermal effect. For example, Millenbaugh et al. (2015) conjugated gold nanoparticles to antibodies specific to *S. aureus* peptidoglycan (Figure 3A). Then, this complex was co-incubated with suspensions of MRSA and methicillin-sensitive *S. aureus* (MSSA) before laser irradiation, which led to significantly decreased bacteria viability and population. Also, Shokri et al. (2015) successfully combined an anti-protein A antibody with polystyrene sulfonate (PSS)-coated gold nanorods (GNRs) to target and kill MRSA through photothermal therapy. Likewise, as for *S. typhimurium* in food samples, nanoparticles with similar designs are also reported frequently (Jeyaraj Pandian et al., 2017). In addition to photothermal sterilization, other enzyme–nanoparticle (NP) conjugates with lysostaphin were proved to be effective (Satishkumar and Vertegel, 2011).

Although antibody just plays a simple role in recognition and binding, it really shrinks the damage range of nanoparticles so as to avoid unnecessary hurt. Nowadays, as monoclonal antibodies harbor higher specificity, affinity, purity, and homogeneity, this combination will show extraordinary prospects in future diagnosis and treatment.

### Peptide-Based Targeted Nanoparticles

Bacteria-targeting peptides can be alternatives to design potent nanoparticles in precise killing. As reported, Hussain et al. (2018) connected vancomycin-loaded nanoparticles with the cyclic 9-amino-acid peptide CARGGLKSC (CARG) which could specifically bind to *S. aureus*, realizing more effective suppression of staphylococcal infections *in vivo* than untargeted treatment (Figure 3B). Moreover, magnetic nanoparticles (MNPs) could also serve as affinity probes to selectively enrich *S. aureus* with peptide HHHHHHDEEGLFVD (D) due to special properties (Kuo et al., 2016). On this basis, improvements lie in the addition of outer package to maintain long effects. For instance, a mesoporous silica nanoparticle (MSN) core was used to transport gentamicin and a cationic human antimicrobial peptide ubiquicidin (UBI29-41) to target the intracellular *S. aureus.* The lipid shell largely prevented the early release of therapeutics (Yang et al., 2018)*.* Likewise, peptides GIBIM-P5S9K (G17) and GAM019 (G19) designed through genetic algorithm optimization strategy and solid-phase chemistry were also reported to be capable of killing MRSA and *E. coli* O157:H7 with a slow and gradual release when encapsulated on poly-lactic-glycolic acid (PLGA) nanoparticles (Gómez-Sequeda et al., 2020).

Sometimes, the combination of nanoparticles and peptides exceeds our initial expectation. For instance, once synthesized peptide HHC10 was conjugated with nanoparticles, it showed potent antimicrobial activity against *E. coli* inside the cells by disintegrating the cell membrane and inducing apoptosis of the host simultaneously (Sharma A. et al., 2018). Also, Esc(1–21), a derivative of the frog skin AMP, targeted the free-living and biofilm forms of *P. aeruginosa* (Casciaro et al., 2017)*.* When it was conjugated to soluble AuNPs, it had 15-fold activity of the free peptide without any toxicity.

These findings are interesting and impressive if we do not take the previous cost of peptide screening into consideration. In this combination, NPs display multiple advantages in effective transport, gradual release, reduced toxicity, and improved potency. However, despite these, the safety of peptide and its concentration control are still noteworthy.

### Membrane-Based Targeted Nanoparticles

Breakthroughs in nanotechnology have contributed to an endless stream of synthetic nanoparticles with supreme specificity since then. Generally, precise delivery is achieved by conjugating nanoparticles with ligands (Hu et al., 2017), peptides (Huang et al., 2017), and antibodies (Lambert and Berkenblit, 2018). However, increasing attention has been paid to the membrane of immune cells recently (Li et al., 2018). A study showed that coating macrophage membrane pretreated with specific bacteria onto the surface of a gold–silver nanocage (GSNC) rendered the nanosystem more efficient (Wang et al., 2018). It was mainly due to the potential interaction between bacterial recognizing receptors on macrophage membranes and distinct pathogen-associated molecular patterns (PAMPs) in bacteria. In another research, Ying et al. (2018) utilized vesicles from the cholesterol-enriched platelet membrane to transport small molecule therapeutics (Figure 3C). Owing to the surface markers on the membrane, this system exhibited natural affinity to both breast cancer cells and MRSA. Meanwhile, compared to RBC-based formulations which support nonspecific improvement of drug bioavailability, platelet membrane–derived vesicles have large potential and distinct advantages for targeted therapy.

Additionally, other natural cell membranes can also be utilized. The membrane of extracellular vesicles secreted by *S. aureus* was reported to be applied to enclose nanoparticles as a natural guide (Gao et al., 2019). This well-designed complex therefore exhibited high specificity and efficacy against *S. aureus* infection in mouse models. For combating *Helicobacter pylori*, biomimetic nanoparticles coated with plasma membranes of gastric epithelial cells could be potent carriers for antibiotics (Angsantikul et al., 2018). They showed inherent adhesion to *H. pylori* bacteria by the same surface antigens as the source cells.

Collectively, all these designs are based on special substances on pretreated membranes, which can produce associations with the target pathogenic bacteria. This is with no doubt an ingenious approach relying on already existed associations.

### Other Specially-Designed Targeted Nanoparticles

Recently, a research showed a brand-new approach to target pathogenic bacteria through the use of functionalized DNA origami nanostructures with aptamers (Mela et al., 2020). Using direct stochastic optical reconstruction microscopy (dSTORM) and atomic force microscopy (AFM), this special structure was confirmed to be capable of binding with and killing bacteria more efficiently through the lysozyme delivered on it than free lysozyme. This research indeed appeared as an innovation and offered us a potent tool in the fight against pathogenic bacteria and the following antibiotic resistance.

To sum up, both metal nanoparticles with photothermal effect and organic nanoparticles as excellent carriers exhibit large potential in precise treatment. The most attractive point lies in the nonexistence of resistance as their intrinsic properties are unique and natural. However, despite their advantages in terms of pharmacokinetics, we should admit that there are still not enough objective judgments for the safety of them, such as systemic toxicity, cytotoxicity, and influence on the blood–brain barrier.

## Phage-Based Targeted Therapy

Phage is one kind of virus that is specific to bacteria, which is abundant in our environment. At the same time, phage therapy has raised much concern since the start of the 20th century in tackling with thorny infections with its personalized characteristics. Although it seems like a novel alternative strategy against antibiotic-resistant bacteria, phages were first used in children’s hospital soon after being reported by Frederick Twort in 1915 in London (Twort, 1915). Compared to traditional antibiotics, phages exhibit several advantages as they can regulate secretion at infection sites through *in situ* multiplication and automatic clearance (Abedon, 2011). Also, phages have minimal possibility to generate resistance as they can coevolve with the host. Notably, it has been widely acknowledged that sublethal levels of some specific antibiotics could enhance the effect of phages regardless of the resistance state. While on the other hand, defects still exist such as the activation of immune response (Nungester and Watrous, 1934), incapability in treating intracellular infections, and the subsequent bacterial resistance (Mahony et al., 2008). However, despite these factors, phage therapy has indeed made some progress in combating VRE, β-lactam–resistant *Enterobacteriaceae*, and MRSA, exhibiting its broad prospects in the targeted therapy (Sybesma et al., 2018).

### Phage Therapy

Phage therapy, the strategy to combat with infectious bacteria by single phage or phage combination, has received outstanding achievements against a series of bacteria.

As for *S. aureus*, bacteriophage and its product such as phi 812 and AB-SA01 exhibited extensive killing ability among hundreds of strains and strong potency *in vitro*, respectively (Pantůcek et al., 1998; Lehman et al., 2019). Sb-1 was another promising phage as it avoided the risk of amputation for patient with diabetic foot ulcers infected with MRSA (Fish et al., 2016). Meanwhile, phage cocktails are effective as mixtures of only six different phages could tackle with the most common *S. aureus* strains (Kelly et al., 2011). Additionally, a phage cocktail of *S. aureus*, CT-SA, can potently eliminate *S. aureus* forming biofilm from chronic rhinosinusitis patients (Drilling et al., 2014). Another research on the treatment of *S. aureus*–induced mastitis also tested the efficacy of the phage cocktail composed by two phages, that is, vBSM-A1 and vBSP-A2, which was comparable to that produced by ceftiofur sodium (Geng et al., 2020).

Another troublesome pathogen is *A. baumannii*, which was classified within the most dangerous critical priority group of MDR bacteria by the WHO in 2017. In face of this urgent circumstance, phage therapy can be an alternative strategy to fill in the vacancy of potent antibiotics. Representatives were Acibel004 and Acibel007, the bacteriophage Loki, phage vB-GEC_Ab-M-G7 (phi G7), and Phage IME200 (Turner et al., 2017; Liu et al., 2019). In clinical cases, phage cocktail was successfully employed by Schooley et al. (2017) to treat diabetic patients with MDR *A. baumannii* infections (Schooley et al., 2017). A mouse infected wound model also highlighted the efficacy of a five-member cocktail of wild phages against *A. baumannii*. Interestingly, these phages seemed to work in a combinatorial manner as one constituent phage transformed capsulated *A. baumannii* into uncapsulated state. This resulted in the enhanced sensibility of *A. baumannii* to the remaining four phages (Regeimbal et al., 2016).

Then, as one of the most common opportunistic pathogens involved in nosocomial infection, *P. aeruginosa* also has its characteristic phage therapies (Driscoll et al., 2007). In a research, Waters et al. (2017) reported the efficacy of phage therapy toward a natural long-term chronic lung infection established by *P. aeruginosa*. Notably, another anti–*P. aeruginosa* phage OMKO1, utilizing the outer membrane porin M (OprM) of the multidrug efflux systems MexAB and MexXY as its receptor-binding site, could target pathogens and avoid resistance simultaneously (Chan et al., 2016). This with no doubt provided an improvement in phage therapy where phages exerted selection for MDR bacteria, allowing the resuscitation of antibiotics with less effectiveness originally. Apart from these examples described above, phage therapy also unfolded its capabilities in other bacteria such as *Mycobacterium abscessus* (Dedrick et al., 2019), as well as *E. coli* and *Proteus* spp (Bernasconi et al., 2017).

### Combination of Antibiotic and Phage

Recently, numerous evidences have sprung up and uncovered the evident advantages between antibiotics and phages than their single use (Chhibber et al., 2013; Kamal and Dennis, 2015). Accordingly, intrinsic antibiotic resistance is tightly associated with properties such as low drug permeability, extrusion of efflux pumps, and expression of SOS systems. Hence, phages with receptors on the membrane-exposed regions of an efflux pump system or overexpressing an SOS suppressor may act as adapters and enhancers of appropriate antibiotics in efficient killing (Chan et al., 2016). Then, a concept called phage-antibiotic synergy (PAS) effect was put forward; that is, sublethal concentrations of antibiotics could contribute to enhancement of the infectivity of phages (Comeau et al., 2007). In this case, an altered physiological state of the host caused cellular filamentation, which led to increased biosynthetic capacity of phages and external attachment to the bacterium. Recently, Shlezinger et al. (2019) in their research illustrated that the efficacy of the combined treatment of vancomycin and phage EFLK1 against VRE cultures was evidently improved compared to single-drug therapy. Also, in an effort to find out the PAS against *P. aeruginosa in vitro*, phage KPP22 was sorted out due to its PAS with a variety of antibiotics and several anti-*Pseudomonas* drugs, particularly piperacillin and ceftazidime (Uchiyama et al., 2018). Explanations based on Darwinian evolution illustrate the logic beneath the positive interactions (Torres-Barceló and Hochberg, 2016). First, genetic constraints or the costs force the bacteria to weigh between resistance mechanisms. Second, the direct negative interaction between resistance mechanisms impedes the evolution of resistance. Third, it is just the low bacterial densities that reduce the possibility of the appearance of resistance mutations.

## CRISPR-Cas9 Technology–Based Targeted Therapy

**FIGURE 4:**
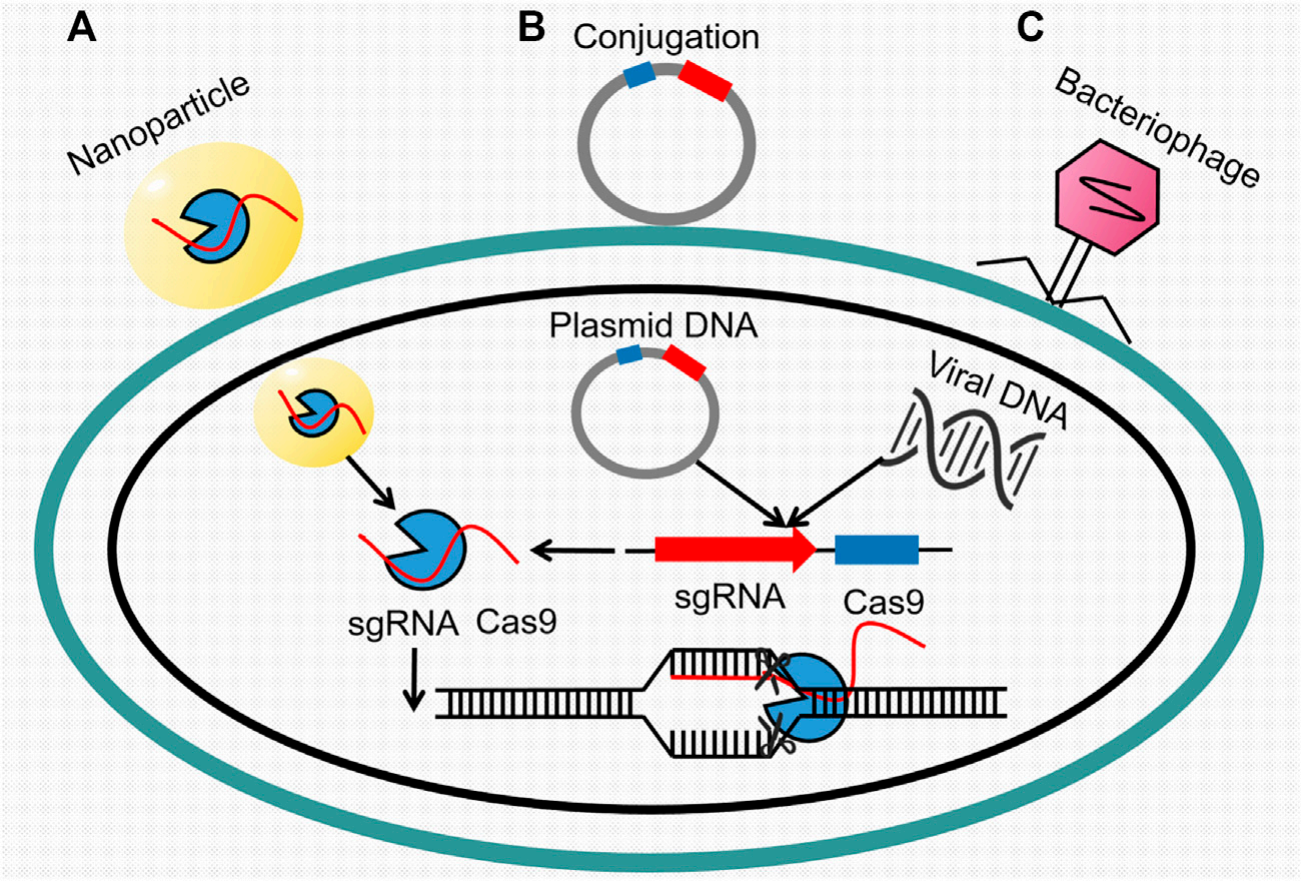
Transport of the CRISPR Cas9 system through three approaches. **(A)** Nanoparticles carrying single-guide RNA and Cas9 protein, **(B)** plasmid DNA through conjugation, and **(C)** viral DNA through bacteriophage invasion make up the main sources of the acquired Cas9 system in target bacterial cell. Thereafter, sgRNA recognizes the specific resistance gene segments in chromosomal DNA, and Cas9 protein conducts cutting activity.

CRISPR-Cas9 system, a protective barrier of acquired immunity found in numerous bacteria and archaea, has received much attention since it was discovered (Doudna and Charpentier, 2014). It includes a CRISPR loci with short repeated sequences (repeats) and similarly sized flanking sequences (spacers), as well as genes encoding Cas-related proteins. These spacers, usually foreign DNA or RNA element, will be inserted in the loci and then be used as guides for Cas protein to perform cutting capability during next invasive incident with the same sequence. Of all the CRISPR-Cas9 systems, Type Ⅱ CRISPR-Cas9 system is one of the best described and exhibits non-ignorable potential in gene editing (Wang et al., 2016). Also, it has been regarded as a precise approach to overcome bacterial infections by selectively targeting genes such as biofilm formation and virulence (De La Fuente-Núñ ez and Lu, 2017). Despite its broad prospects, Type Ⅱ CRISPR-Cas9 system still encounters a large barrier in its further development, that is, the lack of efficient delivery vector. However, this bottleneck has been broken through the development of nanotechnology, genetic recombination, and engineered phages (Figure 4).

In research of Rodrigues, the CRISPR-Cas system was adapted into a constitutively expressed module encoded on a pheromone-responsive conjugative plasmid. It was subsequently transferred to *E. faecalis* for the selective removal of antibiotic resistance genes (Rodrigues et al., 2019). A similar experiment conducted by Dong et al. (2019) also removed the plasmid harboring *mcr-1* effectively. Additionally, Kim et al. (2018) succeeded in searching for a conserved target sequence for CRISPR/Cas9 among numerous ESBL mutants. And the later constructed conjugative plasmids expressing both Cas9 protein and sgRNA could resensitize MDR *E. coli* whose resistance was determined by other genes.

As the alternative, phage-delivered CRISPR targeting also attaches much attention. Liu et al. (2020) integrated the CRISPR/Cas9 system into the genome of a lysogenic phage through novel cloning methods. Then, a phage-delivered resistance eradication with a subsequent antibiotic treatment (PRESA) strategy came out. It displayed multiple advantages such as high plasmid-clearance efficacy, constant inhibition effect, and little or no mutation frequency. Likewise, combining the CRISPR/Cas9 system with a temperate phage, Park et al. (2017) successfully designed a potent weapon with an extended host range of *S. aureus* by eliminating major virulence genes in chromosome. Work of Cobb et al. (2019) achieved similar results against infections caused by *S. aureus* producing biofilm.

Another option is the involvement of nanotechnology. For example, Kang et al. designed a polymer-derived Cas9, which was then combined with targeted single-guide RNA to generate nanosized CRISPR complexes. This complex could target *mecA*, the major gene associated with methicillin resistance of MRSA (Kang et al., 2017). The discovery of CRISPR-Cas technology is really a milestone event in history of genomic editing. Meanwhile, it is still a long march before applying it to clinical treatment even its delivery has been largely improved. What we should seriously consider is how to deal with the more complex gene editing, how to decrease the “off-target” mutations, and most importantly, how to shrink the high cost.

## Challenges and Outlooks

Throughout the context, we put our emphasis on five mainstream antimicrobial strategies and exemplify their feasibility and effectiveness. However, despite their great prospects, obstacles that prevent them from entering into clinic and getting growing popularity are still assignable.

Narrow-spectrum antimicrobials meet their bottlenecks due to the frequent single-target resistance mutations and the risk of prescriptions with precise agents to save patients under critical conditions considering the lag in contemporary pathogen diagnosis and antibacterial spectrum determination. As for bacteriophages, large-scaled sequencing in the early stage requires substantial front input. The clearance by innate immune system directly affects the phage pharmacokinetics. Besides, difficulties in penetrating into mammalian cells, uncertainty in safety, and the increasing resistance are indeed confusing hindrances (Oechslin, 2018). Relatively speaking, probiotics are much safer, but this kind of safety differs from bacteria to bacteria, person to person, and quantity to quantity. Longtime use of artificial probiotics inevitably induces dependence so that our gut will gradually lose the ability to generate its own probiotics. The abuse of probiotic supplements also results in side effects such as infections, metabolic disorder, and abnormal immune functions (Suez et al., 2019). Nanoparticles come to people’s attention with excellent performance, while their syntheses have high standards, which are not appropriate for extensive industrial production. Also, uncertainties in terms of storage, dispersion, stability, cytotoxicity, and degradation are huge hindrances in the way of their advancements. Last but not least, CRISPR-Cas technology is reliable, but sometimes, the “off-target” phenomenon will happen, which largely affects gene editing efficiency (Pursey et al., 2018).

Hopefully, some improvements have been put forward to overcome these challenges. For instance, the application of nucleic acid or mass spectrometry–based technologies greatly accelerates the diagnosis of pathogens prior to prescription of narrow-spectrum antimicrobials. Also, in order to avoid mutations induced by genetic modification during culturing process before administration, inducible repression systems are well designed in engineered probiotics to regulate the expression of functional genes. As for bacteriophages, encapsulating the phage in liposomes and nonimmunogenic polyethylene glycol can improve its *in vivo* stability against the acidic and proteolytic environment and escape from the elimination of the innate immune system, respectively (Kim et al., 2008). Then, the drawbacks of CRISPR-Cas technology have been tackled by target multiplexing and tailored CRISPR-Cas delivery.

All along, what numerous scientists focus on is to avoid the terrible influence of bacterial pathogenicity and subsequent resistance as their combinations may cause incredible bad results. For example, Europe in May 2011 witnessed the outbreak of hemolytic uremic syndrome related to *E. coli* serotype O104:H4, leaving thousands of people under threats (Buchholz et al., 2011). So in our endeavor to fight against pathogenic bacteria, we should be on guard against the potential evolution of resistance. Comfortingly, as we described above, the appropriate combinations of phages and antibiotics well restore the efficacy of antibiotics, and the PAS effect under sublethal concentrations of antibiotics heavily enhance the infectivity of phages. Additionally, engineered probiotics utilizing quorum sensing to realize targeted killing add minimal burden to the host due to their commensal relationship, which circumvents the emergence of resistance. Also noteworthy are nanoparticles, as they hardly induce resistance due to their natural physical properties.

## Conclusions

**FIGURE 5:**
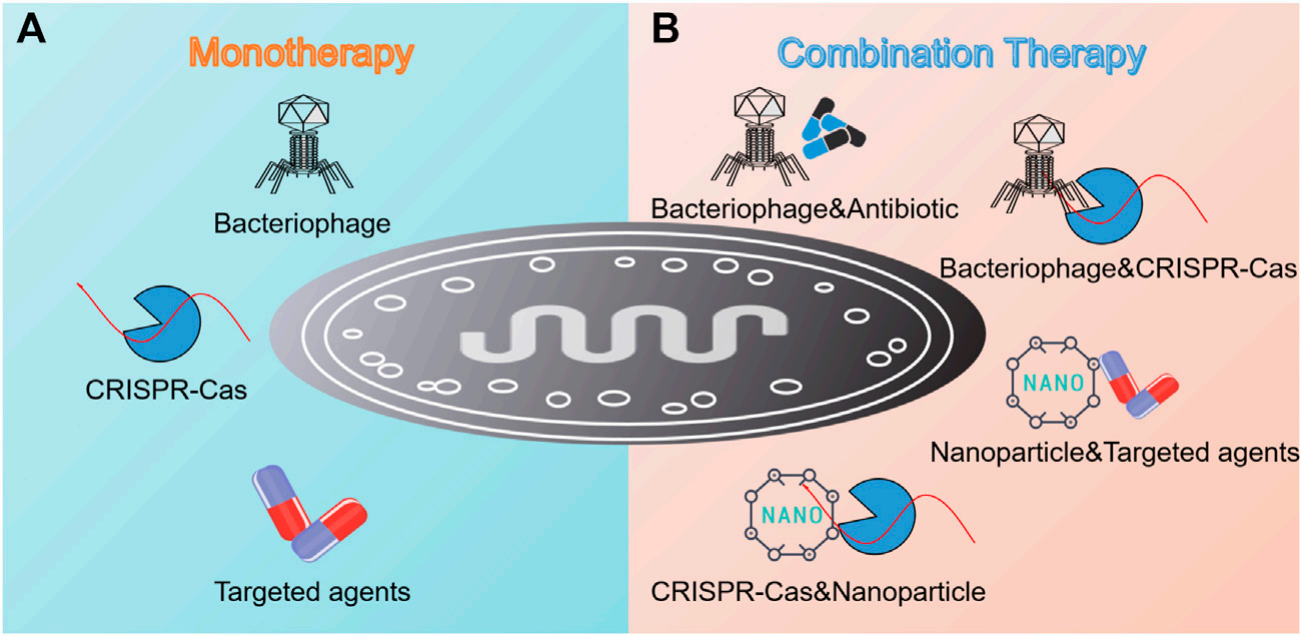
Summary of targeted monotherapy and combination therapy against pathogenic bacteria. **(A)** Monotherapy involves the invasion of bacteriophage, the employment of CRISPR-Cas technology, and the utilization of targeted antimicrobial agents such as antibiotics, lysins, antibodies, and AMPs. **(B)** Combination therapy shows its advantages as reflected in the combination of bacteriophage and antibiotic, bacteriophage and CRISPR-Cas technology, nanoparticles and targeted antimicrobial agents, and CRISPR-Cas technology and nanoparticles.

The increasing resistance crisis calls for novel antimicrobial pipelines, particularly for targeted therapeutic strategies. In this review, an overview of five promising targeted regimens including narrow-spectrum agents, engineered probiotics, nanotechnology, phage therapy, and CRISPR-Cas9 technology was provided. In addition, antibody is another example we have not illustrated sufficiently in the context, but it is of the same weight as others. Until recently, several antibodies against *Staphylococci*, *P. aeruginosa*, *B. anthracis*, and *C. difficile* have been approved for clinical use or are still in different phases of Clinical Efficacy Testing (Table 2). In terms of all strategies mentioned above, they can be divided into two main categories, that is, monotherapy and combination therapy (Figure 5). Usually, the efficacy of the latter exceeds that of the former due to high delivery efficiency, enough local concentration, synergistic effect, and low resistance frequency.

**TABLE 2:** Typical antibodies against four pathogenic bacteria in drug development.

Antibodies	Pathogens	Targets	Company	Clinical studied	References
Raxibacumab	*B. anthracis*	Protective antigen	GlaxoSmith Kline	FDA approved	Subramanian et al. (2005)
Obiltoxaximab	*B. anthracis*	Protective antigen	Elusys Therapeutics	FDA approved	Hou and Morrill (2017)
Bezlotoxumab	*C. difficile*	Toxin B	Merck & Co.	Phase III	Wilcox et al. (2017)
Actoxumab	*C. difficile*	Toxin A	Merck & Co.	Phase III	Hernandez et al. (2017)
Panobacumab	*P. aeruginosa*	LPS O antigen (O11)	Kenta Biotech	Phase II/III	Lazar et al. (2009)
MEDI-3902	*P. aeruginosa*	PcrV type III secretion system (T3SS) and persistence factor Psl	MedImmune	Phase II	Ali et al. (2015)
Anti-pseudomonas IgY	*P. aeruginosa*	Immunsystem AB	Unknown	Phase I/II	Thomsen et al. (2016)
MEDI-4893	*Staphylococci*	Alpha toxin	MedImmune	Phase II	Yu et al. (2017)
Pagibaximab	*Staphylococci*	Lipoteichoic acid	Biosynexus	Phase III	Patel and Kaufman (2015)
Aurexis	*Staphylococci*	Clumping factor A	Bristol-Meyrs Squibb	Phase II	Weems et al. (2006)

Nevertheless, it makes great sense to deepen the current knowledge about these innovative therapeutic strategies because they are not mature, stable, and reliable enough at present. Although these endeavors would be time-consuming, labor-intensive, and probably limited to laboratories, they are indeed meaningful as currently effective antimicrobials will end up losing their activities someday. Considering that antibiotic resistance has become a global concern, we firmly believe that with the advancement of technology, great progress will be made in these works.
